# Innovative Conservative Management of a Mid-root Fracture in a Maxillary Lateral Incisor: A Case Report

**DOI:** 10.7759/cureus.115050

**Published:** 2026-08-23

**Authors:** Sanjeev Srivastava, Chirantan Chowdhury, Arohan Singh, Shubhangi Shubhangi

**Affiliations:** 1 Department of Conservative Dentistry and Endodontics, Sardar Patel Post Graduate Institute of Dental and Medical Sciences, Lucknow, IND

**Keywords:** bioactive glass, conservative endodontic management, dental trauma, fiber post, horizontal root fracture, interfragmentary healing, maxillary central incisor, richmond crown

## Abstract

A 22-year-old male patient presented with trauma-induced injury to the maxillary anterior teeth following a road traffic accident. Clinical and radiographic examination revealed a middle-third horizontal root fracture with pulpal necrosis in the maxillary lateral incisor and a complicated crown fracture with symptomatic irreversible pulpitis in the maxillary central incisor. As the patient declined surgical intervention, a conservative multidisciplinary treatment approach was planned. The fractured root was repositioned and stabilized using a semi-rigid splint followed by non-surgical endodontic treatment. After sectional obturation, the fracture interface was managed using orthograde placement of bioactive glass slurry with intermittent ultrasonic activation to enhance penetration and adaptation within the interfragmentary space. A fiber post luted with dual-cure composite resin was subsequently used to reinforce and stabilize the fractured segments. The adjacent central incisor was treated endodontically and restored using an all-ceramic Richmond crown because of extensive coronal tooth structure loss. Clinical and radiographic follow-up demonstrated satisfactory stabilization, preservation of periodontal integrity, favorable healing, and acceptable esthetic rehabilitation. The present case highlights the potential of bioactive glass-assisted conservative management as a minimally invasive strategy for the treatment of middle-third horizontal root fractures while simultaneously restoring function and esthetics in severely compromised anterior teeth.

## Introduction

Traumatic dental injuries to the permanent dentition are prevalent among children and young adults, with the maxillary anterior teeth being particularly vulnerable because of their labial prominence and exposure to direct impact. Among the different types of traumatic dental injuries, crown fractures are the most prevalent. These types of fractures may involve only enamel, enamel and dentin (uncomplicated fracture), or may extend to the pulp (complicated crown fracture) [1].

In contrast, horizontal root fractures represent a relatively uncommon form of dental trauma, accounting for approximately 0.5%-7% of traumatic injuries in permanent teeth. They are classified according to the location of the fracture line into apical-, middle-, and cervical-third fractures, with middle-third fractures being the most frequently reported in the literature [2]. The biological response and prognosis of horizontally fractured teeth are influenced by multiple variables, including the stage of root development, degree of displacement and separation (diastasis) between fragments, pulpal status, and the timeliness and adequacy of long-term repositioning and approximation of the fragments. Long-term stabilization and approximation of the coronal fragment remain critical determinants of successful clinical outcomes [2].

A significant biological challenge associated with horizontal root fractures is the presence of an interfragmentary space at the fracture interface. This space may serve as a potential ecological niche for microbial colonization, particularly in cases in which pulpal necrosis develops within the coronal segment. Bacterial contamination of the fracture interface can interfere with tissue repair processes, leading to inflammatory root resorption or persistent periradicular pathology [3]. Therefore, successful management necessitates not only thorough chemomechanical debridement of the infected canal system but also effective sealing or obliteration of the fracture interface to eliminate microbial reservoirs and facilitate favorable healing. Although surgical approaches, including flap reflection and direct management of the fracture site, have been advocated in selected cases, such interventions are invasive and may compromise periodontal attachment, crown-root ratio, and long-term prognosis. The advent of bioactive materials has expanded conservative treatment strategies. Bioactive glass, characterized by its biocompatibility and capacity to induce hydroxyapatite formation through ionic exchange, promotes mineralization and hard tissue deposition while creating a local environment unfavorable to microbial survival [4-6]. Orthograde placement of bioactive material at the fracture interface represents a minimally invasive approach aimed at sealing the interfragmentary space and enhancing biological repair around the fractured segments.

This case report describes the conservative management of a middle-third horizontal root fracture using nonsurgical endodontic therapy supplemented with orthograde application of bioactive glass to optimize interfragmentary healing and clinical prognosis.

## Case presentation

A 22-year-old male patient reported to the Department of Conservative Dentistry and Endodontics with the chief complaint of broken and mobile upper front teeth following trauma sustained in a road traffic accident five days earlier. Intraoral examination revealed grade I mobility in relation to tooth #12, with no associated swelling or sinus tract formation, and the surrounding soft tissues appeared normal. Tooth #12 was nontender to percussion. On cold vitality testing, no response was elicited from tooth #12, whereas tooth #11 exhibited an exaggerated and lingering response. Electric pulp testing (EPT) revealed no response in relation to tooth #12, while tooth #11 showed an exaggerated response.

Intraoral periapical radiographic examination revealed a horizontal fracture involving the middle third of the root extending from the mesial to distal aspect in relation to tooth #12, along with a complicated crown fracture involving enamel, dentin, and pulp in relation to tooth #11. Based on the combined clinical and radiographic evaluation, the final diagnosis was pulpal necrosis associated with a middle-third horizontal root fracture in relation to tooth #12 and symptomatic irreversible pulpitis secondary to a complicated crown fracture in relation to tooth #11. As the patient was apprehensive about undergoing surgical intervention, a conservative treatment plan was formulated, which involved nonsurgical endodontic treatment for tooth #12, followed by reattachment of the fractured segment, and nonsurgical endodontic therapy for tooth #11, followed by rehabilitation with a Richmond crown.

**Figure 1:**
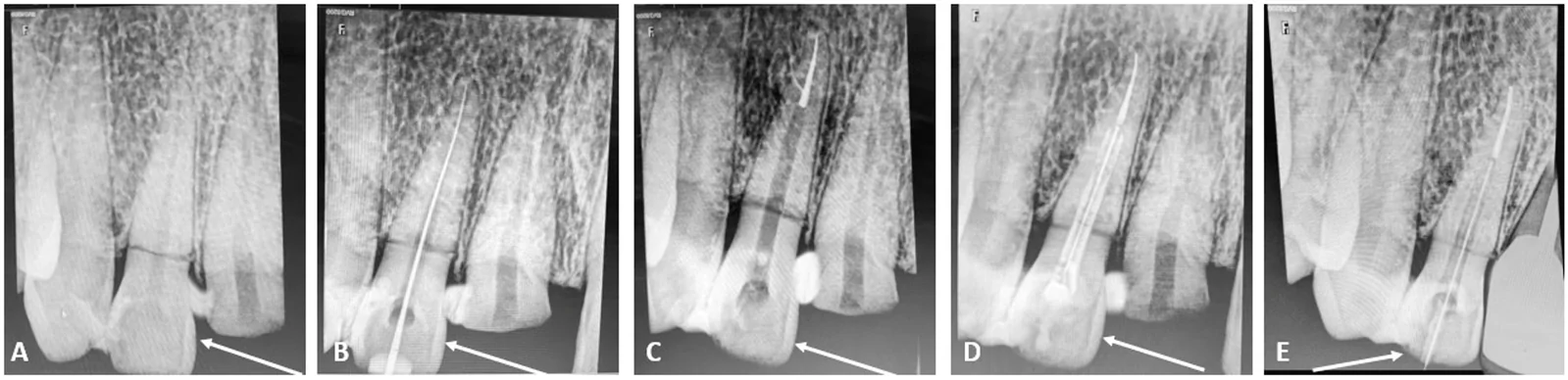
Radiographic sequence illustrating the conservative management of a middle-third horizontal root fracture in tooth #12 using fiber post stabilization A) Preoperative radiograph showing repositioning of the fractured fragment and stabilization with a semirigid splint; B) working length determination following access opening and biomechanical preparation; C) post-space preparation performed while maintaining an adequate apical seal after sectional obturation; D) placement and cementation of the prefabricated fiber post using dual-cure resin cement for intraradicular stabilization of the fractured fragments; E) a seven-month follow-up radiograph demonstrating satisfactory healing and stability of the fractured segments. The arrow indicates the treated tooth.

After obtaining written informed consent, at the first appointment, the fractured fragment in relation to tooth #12 was repositioned and stabilized using a semirigid splint (Figure 1A). Subsequently, access opening was performed under rubber dam isolation. Working length was determined using an electronic apex locator (Root ZX; J. Morita Corp., Kyoto, Japan) and verified radiographically (Figure 1B). Cleaning and shaping are performed using rotary nickel-titanium files (ProTaper Gold; Dentsply Sirona, Ballaigues, Switzerland) up to size F3 (30/.09) with copious irrigation using 2% chlorhexidine and 17% EDTA. Sectional obturation of tooth #12 was completed using gutta-percha in conjunction with an epoxy resin-based sealer (AH Plus; Dentsply Sirona, Konstanz, Germany).

At the subsequent visit two days later, post-space preparation was carried out while preserving an adequate apical seal (Figure 1C). The prepared post space was irrigated with a slurry of bioactive glass and ultrasonically activated for three cycles of 10 seconds each to facilitate penetration and adaptation. This was followed by irrigation with normal saline to remove excess bioactive glass slurry, and the canal was dried with sterile paper points.

A prefabricated fiber post was tried in to verify appropriate fit and length. The canal walls were treated with a self-curing adhesive system (ParaBond; Coltene, Altstätten, Switzerland) using a microbrush for 30 seconds, after which excess material was removed with paper points and the post space was gently air-dried. ParaCore dual-cure core build-up material (ParaCore; Coltene, Altstätten, Switzerland) was then injected directly into the canal using an automix tip, and the fiber post was immediately seated. Excess material was removed, and light curing was performed for 20 seconds to ensure adequate polymerization and stabilization of the fractured fragments (Figure 1D). At the seven-month follow-up, the patient remained asymptomatic, with no evidence of pain, mobility, or tenderness to percussion and palpation. Radiographic evaluation demonstrated satisfactory alignment and stabilization of the fractured segments, with maintenance of the periodontal ligament space and absence of periapical radiolucency or inflammatory root resorption. Evidence of hard tissue healing was observed at the fracture line, indicating favorable healing and successful functional stabilization achieved through the intraradicular fiber post-reinforcement approach (Figure 1E).

**Figure 2:**
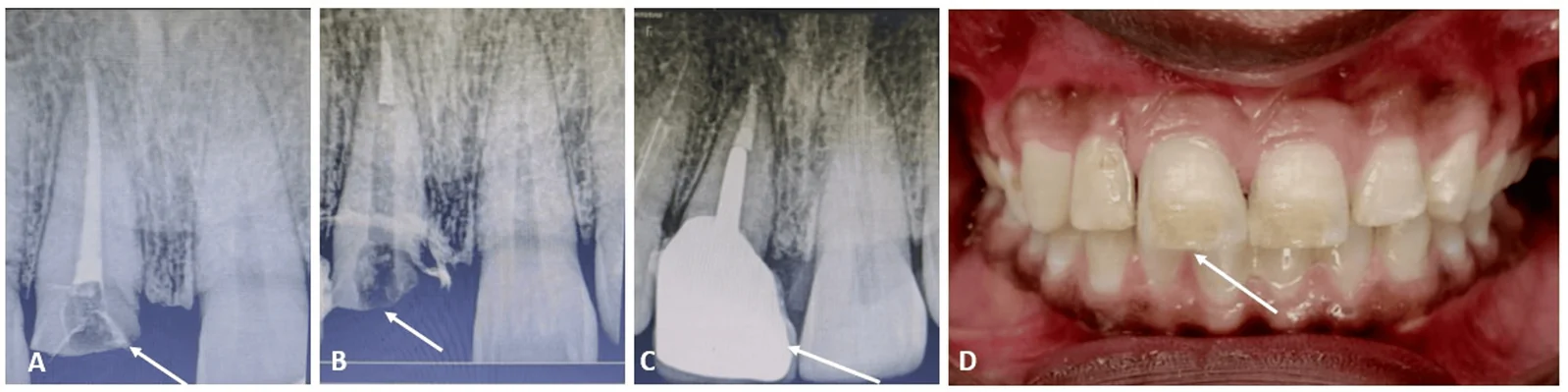
Clinical and radiographic sequence illustrating the post-endodontic rehabilitation of tooth #11 using an all-ceramic Richmond crown A) Obturation completed with gutta-percha and AH Plus sealer following biomechanical preparation; B) post-space preparation performed while preserving 4 mm of apical gutta-percha to maintain the apical seal; C) radiographic verification of the fit and adaptation of the Richmond crown during the try-in appointment; D) postoperative clinical photograph showing the cemented all-ceramic Richmond crown with satisfactory esthetic and functional rehabilitation. The arrow indicates the treated tooth.

Five days later, endodontic treatment was initiated in relation to tooth #11 under rubber dam isolation. Cleaning and shaping were performed using rotary nickel-titanium files (ProTaper Gold; Dentsply Sirona, Ballaigues, Switzerland) up to size F3 (30/.09), along with irrigation using 5.25% sodium hypochlorite and 17% EDTA. Calcium hydroxide intracanal medicament was placed, and the patient was recalled after 5 days. Obturation was completed using gutta-percha and AH Plus sealer (Figure 2A). Post-space preparation was carried out while preserving 4 mm of apical gutta-percha (Figure 2B).

Tooth preparation was performed for a Richmond crown. Using a J-hook made from 22-gauge stainless steel wire and light-body elastomeric impression material (Aquasil; Dentsply Sirona, Milford, DE, USA), a final impression was made using addition silicone impression material. The impression was digitally scanned, and an all-ceramic Richmond crown was fabricated. At the try-in appointment, radiographic evaluation confirmed satisfactory fit (Figure 2C). The crown was cemented using a self-curing resin-based luting cement (Multilink N; Ivoclar Vivadent, Schaan, Liechtenstein) according to the manufacturer’s instructions (Figure 2D).

## Discussion

Root fracture can occur as a consequence of dental trauma, resulting in complex injury to the cementum, dentin, pulp, and periodontal tissues. Horizontal root fractures are relatively uncommon and present significant therapeutic challenges, with treatment strategies largely determined by the location and extent of the fracture. The most challenging aspect of treatment is the approximation and stabilization of the fractured fragments. A critical determinant of prognosis is the position of the fracture line. Apical-third fractures generally exhibit the best prognosis because of minimal mobility of the coronal fragment and better preservation of pulpal blood supply, which promotes favorable healing. Middle-third fractures have a comparatively moderate prognosis, whereas coronal (cervical)-third fractures demonstrate the poorest prognosis because of increased mobility, greater periodontal involvement, and a higher risk of pulpal necrosis [1-2].

Middle-third root fractures are frequently associated with biological and mechanical complications secondary to disruption of pulpal and periodontal integrity. These complications may include pulpal necrosis of the coronal fragment, inflammatory root resorption, and periapical pathology in cases of microbial contamination. Achieving optimal approximation and stabilization of the fracture fragments remains the most challenging aspect of management [2].

Although surgical intervention involving elevation of a mucoperiosteal flap may provide direct access for fracture stabilization, this approach is inherently invasive and may lead to postoperative pain, swelling, discomfort, additional alveolar bone loss, periodontal attachment compromise, and delayed healing. The introduction of bioactive materials such as bioactive glass has enabled a more biologically driven and minimally invasive treatment alternative. Sauro et al. reported that bioactive glass exhibits osteoinductive and osteoconductive properties. Upon contact with tissue fluids, it releases calcium and phosphate ions that promote the formation of a hydroxyapatite layer on the dentin surface. This apatite layer closely resembles the mineral phase of dentin and bone, facilitating chemical bonding with adjacent hard tissues and enhancing mineralized tissue deposition at the fracture interface [6]. By facilitating hard tissue deposition between the fractured segments, bioactive glass aids in the subsequent obliteration of the intersegmental dead space, thereby enhancing fragment approximation and promoting favorable healing [4,6].

Consequently, a conservative fracture repair strategy was implemented to preserve periodontal integrity and promote optimal biological healing while avoiding surgical trauma.

In the present case, the bioactive glass slurry was ultrasonically activated for three cycles of 10 seconds each. Intermittent ultrasonic activation enhances the material's penetration and adaptation into the intersegmental space, reduces void formation, and ensures uniform distribution along the fracture interface. Moreover, Barbhai et al. concluded that controlled cyclic activation facilitates optimal ionic release, which is necessary for hydroxyapatite formation, while minimizing thermal injury to surrounding periodontal tissues and maintaining material stability [7].

A fiber post was selected for intracanal reinforcement because its modulus of elasticity closely approximates that of dentin, thereby promoting uniform stress distribution along the root and reducing the risk of secondary or catastrophic fracture, as reported by Ferrari et al. [8]. In contrast to rigid metallic posts, fiber posts bonded with composite resin create a favorable monoblock effect, enhancing the structural integrity of the reattached segments. Additionally, fiber posts require minimal canal preparation, thereby preserving tooth structure and maintaining root strength. Their translucency is particularly advantageous in anterior teeth, as it improves light transmission and contributes to superior esthetic integration with definitive restorations.

In the subsequent phase of treatment, the maxillary central incisor exhibited a complicated crown fracture with greater than 75% loss of coronal tooth structure. The remaining dentinal walls were insufficient to provide adequate retention and resistance form for a conventional post-and-core system followed by a separate full-coverage crown, and an ideal ferrule could not be predictably achieved. Therefore, a Richmond crown was planned as the definitive restorative modality [7-8].

The Richmond crown is a single-piece custom restoration in which the post, core, and crown are fabricated as a unified structure. This monoblock design enhances retention and resistance in severely compromised endodontically treated teeth and eliminates the post-core interface, thereby reducing the risk of core dislodgement and mechanical failure. Studies evaluating cast post-and-core restorations have reported that a one-piece design provides superior retention and structural integrity in teeth with extensive coronal destruction compared with prefabricated post systems [8].

Compared with a conventional metal post and core restored with a porcelain-fused-to-metal crown, an all-ceramic Richmond crown provides superior translucency, eliminates metal shadowing and gingival discoloration, and offers better biocompatibility, resulting in enhanced esthetic and long-term clinical outcomes [7-8].

## Conclusions

The present case demonstrates that conservative management of middle-third horizontal root fractures using orthograde placement of bioactive glass can effectively promote interfragmentary healing while preserving periodontal integrity. Ultrasonic activation of the bioactive glass slurry facilitated enhanced penetration and uniform adaptation within the fracture interface, thereby supporting mineralized tissue deposition and stabilization of the fractured segments. The use of a fiber post further reinforced the root by promoting favorable stress distribution, while rehabilitation of the tooth with a complicated crown fracture using an all-ceramic Richmond crown restored both function and esthetics in a severely compromised anterior tooth, resulting in a favorable clinical outcome.
